# The Operation Theatre

**Published:** 1909-10-30

**Authors:** 


### THE OPERATION THEATRE.

### IV.—FITTINGS.

In the first article dealing with this department we noticed briefly some of the fittings required in the theatre itself. We now propose to describe in detail the fittings indicated on the plan which appeared in our issue of October 16. But first we would point out that in this plan there is an error of drawing which would make it appear that the nurses' room, cloak-room, and wash-up-room are only accessible through the operation-room. This, of course, is not intended; there should be a wide opening from the corridor, where the ambulance stands, into the lobby which gives access to these three rooms.

*Theatre.*—In the theatre itself the only fitting involving a connection with the drainage system is a lavatory basin for the surgeons' use. The basin itself should be of glazed porcelain. No plug or waste valve is needed, as the water will always be running continuously while the basin is in use. The waste-pipe should discharge into a channel in the floor, which in turn should discharge into an open trap, the outgo being connected to a pipe passing through the wall and discharging into a second open trap. If the theatre is on an upper floor the outgo from the trap inside will be connected into a vertical pipe, which should be ventilated by being carried up above the eaves of the roof. To keep the trap inside the theatre always well flushed out a tap should be fixed just over it, and the water allowed to run continuously into the trap during the time the theatre is in use.

The method to be adopted of laying on the hot and cold water to the basin is one which is open to discussion. The point to be aimed at is that the surgeon shall have complete control over the flow and the temperature of the water without having to use his hands to turn taps on or off. This can be done either by the foot or by the knee or by the elbow. Whichever plan is adopted it is necessary that there should be a mixing valve into which the hot and cold water have access at an equal pressure, so that the water can be mixed to any required temperature. This valve must be so arranged that it is impossible to turn on the hot water before the cold. The treadle plan has the advantage that it leaves the arms of the person using the basin entirely free, a point which is certainly of some value. The elbow arrangement is, on the other hand, rather simpler in the matter of pipes and valves. Of the valves actuated by the knee we cannot give an opinion, having never seen them in work. Whatever plan is adopted for opening and closing the valves the outlet over the basin should be a rose, from which the water issues in a finely divided spray.

Next to the basin is fixed a small apparatus for providing sterilised water, either hot or cold. The water is boiled by means of a steam coil, and is cooled down to the required temperature by passing cold water through the jacket.

A hot closet for towels adjoins the water steriliser, and is also heated by steam.

The only other fittings inside the theatre are glass shelves for lotions, bowls, trays, and other things required during operations. The set of shelves on the right should be fixed high enough up to allow of a table being wheeled underneath.

*Sterilising-room.*—The slop sink is a glazed porcelain square sink with a flushing rim and a two-gallon flushing tank; the outgo of this sink must be trapped and connected to a vertical pipe, which must be ventilated and treated in every way as a soil pipe. Two wash-up sinks are placed alongside the slop sink each with a teak draining-board. These sinks must have hot and cold water laid on; the wastes must be trapped and connected to a waste-pipe outside, which must discharge over a trapped gully. The waste-pipe must be ventilated. The instrument steriliser is a small vessel heated by a " steam pad " and fixed on the wall by cast-iron enamelled brackets. The large steriliser on the south wall is an adaptation of Lyon's patent; a steam-jacketed machine in which the drums containing dressings, operating coats, etc., can be sterilised by dry steam under pressure. The two small sterilisers at the side are for boiling bowls, trays, etc.

*Surgeons'-room.*—In this room all that is required is a lavatory basin, which should have a mixing valve and spray fitting, but need not necessarily be fitted with either treadle or elbow gear.

*Anæsthetic-room.*—A small sink with hot and cold water and a cupboard for the needful apparatus used for anæsthetising are all the fittings wanted in this room.

*Nurses' Room.*—In this room a plentiful supply of cupboards for the storage of linen bandages, dressings, bowls, trays, splints, and everything required in the operation theatre must be provided. As this room will be used as a workroom for all purposes connected with the theatre, space is provided for a work table for the nurse in charge.

*Cloak-room.*—A sufficient supply of pegs for coats is all that is needed here.

*Wash-up-room.*—Here is a row of basins for the nurses and house surgeon and dressers, each of which must be fitted in exactly the same way as the basin in the theatre. A set of two glass shelves for the sterilised coats, veils, and overalls must be provided; and in this room the instrument-cabinet will stand.

### HOUSEHOLD APPLIANCES.

We have had the opportunity of inspecting some of the products of the Model Home Specialities Co., Ltd., of Wellington Road, Battersea The company's aim is to supply all those articles of household application that people have now to purchase from a variety of sources. It is felt that in this way a standardisation of quality may be gained which would otherwise be impossible. To give some idea of the comprehensive nature of the Model Home Specialities Co., we may quote the following among other articles they manufacture: Furniture polish, black enamel, ready-mixed paint, disinfectant fluid, an embrocation made solely for the firm, and, lastly, for the purposes of illustration, petrol which drives dirt from every form of clothes as fast as if it were a motor. There must be a large public wanting to purchase "home specialities" not only from one shop, but from *one firm*; it is this public which will appreciate the uniform quality in the goods of the above-mentioned company

